# A comparison of the reparative and angiogenic properties of mesenchymal stem cells derived from the bone marrow of BALB/c and C57/BL6 mice in a model of limb ischemia

**DOI:** 10.1186/scrt245

**Published:** 2013-07-26

**Authors:** Flavia Franco Cunha, Leonardo Martins, Priscila Keiko Matsumoto Martin, Roberta Sessa Stilhano, Sang Won Han

**Affiliations:** 1 Research Center for Gene Therapy, Universidade Federal de São Paulo, São Paulo, SP, Brazil; 2 Department of Biophysics, Universidade Federal de São Paulo, Rua Mirassol 207, São Paulo, SP CEP 04044-010, Brazil

**Keywords:** Angiogenesis, Cell therapy, Hind-limb ischemia, Mesenchymal stem cells

## Abstract

**Introduction:**

BALB/c mice and C57/BL6 mice have different abilities to recover from ischemia. C57/BL6 mice display increased vessel collateralization and vascular endothelial growth factor expression with a consequent rapid recovery from ischemia compared with BALB/c mice. Mesenchymal stem cells (MSCs) are one of the main cell types that contribute to the recovery from ischemia because, among their biological activities, they produce several proangiogenic paracrine factors and differentiate into endothelial cells. The objective of this study was to evaluate whether the MSCs of these two mouse strains have different inductive capacities for recovering ischemic limbs.

**Methods:**

MSCs from these two strains were obtained from the bone marrow, purified and characterized before being used for *in vivo* experiments. Limb ischemia was surgically induced in BALB/c mice, and MSCs were injected on the fifth day. The evolution of limb necrosis was evaluated over the subsequent month. Muscle strength was assessed on the 30th day after the injection, and then the animals were sacrificed to determine the muscle mass and perform histological analyses to detect cellular infiltration, capillary and microvessel densities, fibrosis, necrosis and tissue regeneration.

**Results:**

The MSCs from both strains promoted high level of angiogenesis similarly, resulting in good recovery from ischemia. However, BALB/c MSCs promoted more muscle regeneration (57%) than C57/BL6 MSCs (44%), which was reflected in the increased muscle strength (0.79 N versus 0.45 N).

**Conclusion:**

The different genetic background of MSCs from BALB/c mice and C57/BL6 mice was not a relevant factor in promoting angiogenesis of limb ischemia, because both cells showed a similar angiogenic activity. These cells also showed a potential myogenic effect, but the stronger effect promoted by BALB/c MSCs indicates that the different genetic background of MSCs was more relevant in myogenesis than angiogesis.

## Introduction

Peripheral arterial disease (PAD) obstructs the arteries, leading to decreased blood flow. PAD is a chronic disease that affects approximately 3 to 10% of the population [1]. Currently, PAD is primarily treated through the control of cardiovascular risk factors and the use of antiplatelet drugs, anticoagulants and vasodilators. Mechanical techniques, such as angioplasty and surgical revascularization, are reserved for the more severe cases. Even with these therapeutic techniques, 30% of patients can suffer limb amputation within a year due to the progression of the disease [2]. These patients depend on the adaptation of pre-existing collateral vessels (arteriogenesis) or the formation of new vessels via vasculogenesis or angiogenesis to rescue tissue oxygenation [3]. Therapeutic neovascularization using angiogenic factors or stem cells, aimed at rapidly revascularizing the ischemic area, thus represents a potential treatment option for regenerating the damaged tissue and preventing amputations.

Mesenchymal stem cells (MSCs) have the property of adherence to plastic and the ability to self-renew and differentiate into osteoblasts, chondrocytes and adipocytes [4]. Currently, these cells are known to differentiate into more different cell types, including skeletal muscle [5,6], cardiomyocytes and endothelial cells [7-9]. Besides multipotency, MSCs are capable of suppressing the immune system by secretion of nitric oxide, prostaglandins, indoleamine 2,3-dioxygenase, IL-6 and others, which modulate T cells, natural killer cells, dendritic cells and macrophages [10-12]. These properties have allowed MSCs to be used as an immunosuppressant for transplantations, autoimmune diseases and graft-versus-host disease [13].

Therapy using stem cells and progenitor cells has been widely used in the cardiovascular field, particularly the injection of bone marrow mononuclear cells [14-18] and endothelial progenitor cells [19,20]. However, MSCs seem to be more effective for the treatment of limb ischemia [21]. Several *in vitro* studies [22-24] and *in vivo* studies [7,25-27] indicate that MSCs are an important tool for neovasculogenesis, especially during ischemia, because the reduction in oxygen levels (hypoxia) induces MSCs to form capillary-like structures *in vitro*[28]. When implanted, MSCs differentiate and acquire characteristics of mature endothelial cells [7,29], and they can also differentiate into vascular smooth muscle cells and cardiomyocytes [29,30]. However, one of the greatest potentials of MSCs for neovascularization is related to their trophic effects. MSCs produce cytokines with paracrine effects, can prevent fibrosis and apoptosis, can promote angiogenesis and arteriogenesis and can stimulate the proliferation and differentiation of tissue-specific progenitors, thus contributing to tissue repair [31]. The injection of MSCs from different sources was able to increase blood flow and vascular density in ischemic limbs [7,27,32].

Some studies have shown differences in the vasculature of different strains of mice [33-35]. Chalothorn and colleagues demonstrated that C57/BL6 mice have a greater density of pre-existing collateral vessels in all tissues and a better recovery rate from ischemia compared with BALB/c mice [33]. These differences were due to the increased vessel collateralization, a higher rate of angiogenesis and increased expression of vascular endothelial growth factor (VEGF; baseline and post ischemia) and TNFα in the C57/BL6 mice compared with the BALB/c mice. These differences were due to a polymorphism in the cis-acting gene of VEGF-A of BALB/c mice on chromosome 17 that reduced the transcription and expression of this gene in response to ischemia. These observations lead us to question whether MSCs from different strains will provide a difference in the treatment of ischemia. To address this question, MSCs from C57/BL6 mice and BALB/c mice were injected into the ischemic limbs of BALB/c mice to compare the ability of limb recovery from ischemia.

## Materials and methods

### Isolation and characterization of MSCs from mice bone marrow

Experiments were conducted on 8-week-old BALB/c and C57/BL6 male mice. All procedures were approved by the Research Ethics Committee of the Federal University of São Paulo, Brazil (approval number: CEP 0327/10).

To isolate the MSCs, 8-week-old BALB/c mice and C57/BL6 mice were sacrificed by cervical dislocation and the tibia and femur were dissected, and low-glucose DMEM supplemented with 10% fetal bovine serum, 2 mM l-glutamine, 200 U/ml penicillin, and 200 mg/ml streptomycin (DMEMc medium) was injected into the dissected bone to collect bone marrow cells. These cells were maintained in six-well plates for a few days [36]. Adherent cells were detached with 0.25% trypsin, centrifuged, resuspended and plated in culture bottles (25 or 75 cm^2^) with DMEMc medium at 37°C and with 5% CO_2_. After establishing the primary culture, cells were expanded and maintained until injection. All reagents were obtained from Invitrogen Co. (Sao Paulo, Brazil).

The differentiation capacity of these cells into adipocytes and osteoblasts was evaluated based on an established protocol [37]. For osteogenic differentiation, 2 × 10^5^ cells (at passage 8) were plated per well of a six-well plate. DMEM supplemented with 10^–8^ mol/l dexamethasone, 5 mg/ml ascorbic acid 2-phosphate, 10 mmol/l β-glycerophosphate and 10% fetal bovine serum were used with medium exchange every 3 days for 3 to 4 weeks. The osteoblasts were stained with 2% Alizarin Red S, pH 4.1 (Sigma-Aldrich, St Louis, MO, USA). For adipogenic differentiation, 2 × 10^5^ cells were plated per well of a six-well plate containing DMEM supplemented with 10% fetal bovine serum, 10^–8^ mol/l dexamethasone, 2.5 μg/ml insulin, and 5 mmol/l rosiglitazone. The cells (at passage 8) were maintained with medium exchange every 3 days for 3 to 4 weeks. For staining, the medium was aspirated, and then cells were fixed with 4% paraformaldehyde, washed with PBS and incubated with Oil Red O (3.75% in 60% isopropyl alcohol) for 5 minutes.

### Analysis of vascular endothelial growth factor expression by real-time PCR

RNA was extracted from MSCs using the RNeasy mini kit (Qiagen, Hilden, Germany) and treated with DNAse I (Sigma-Aldrich). The cDNA was synthesized using the High Capacity Reverse Transcription cDNA kit (Life Technologies, Sao Paulo, Brazil) and quantitative RT-PCR assay was performed using SYBR Green QuantiFast RT-PCR kit (Qiagen) in the Rotor Gene-Q (Qiagen). The following primers were used to quantify the murine VEGF level: VEGF_F, AGC CAT CCT CTT CTG CAC TT; and VEGF_R, TGG GAA GAG AGC TGG AGT TT. The relative gene expression was calculated by 2^–ΔΔCT^ method. The murine β-actin gene was used to normalize the data with the following primers: β-actin_F, GCT CCT CCT GAG CGC AAG; and β-actin_R, CAT CTG CTG GAA GGT GGA CA. Each reaction was performed in duplicate, and each experiment was performed three times.

### Induction of hind-limb ischemia and cell therapy

Ischemia was induced surgically in 10-week-old to 12-week-old BALB/c mice. After being anesthetized with ketamine (40 mg/kg) and xylazine (10 mg/kg), ischemia was induced in the right leg by removing the entire femoral artery and closing its branches (deep femoral, epigastric, saphenous and popliteal arteries), based on the procedure already established in our laboratory [38,39].

Five days after ischemic induction, the mice were anesthetized again, the quadriceps muscles were exposed and 5 × 10^5^ cells in 50 μl serum-free DMEM were injected into the middle of the muscle using a 21 G needle. MSCs with passage numbers between 7 and 10 were used in the *in vivo* experiments.

The mice were divided into the following groups (*n* = 6 per group): nonischemic animals (N-IS), sham-operated animals (S), ischemic untreated animals (IS), ischemic animals treated with MSCs obtained from BALB/c mice (MSC-Ba) and ischemic animals treated with MSCs obtained from C57/BL6 mice (MSC-Bl).

### Visual assessment and determination of muscle force

The animals were followed-up for 35 days, and a visual assessment of the limbs was performed weekly based on the following scale: I, no change; II, nail-blackening; III, necrosis on toes; and IV, necrosis below the heel.

On the 35th day, before the euthanasia of animals, the isometric muscle force was determined according to the method standardized in our laboratory [38,39]. Briefly, the mouse was anesthetized, the gastrocnemius muscle was isolated completely while maintaining the vascular connections and origin of the muscle, and the tendinous insertion of the muscle was isolated and bound to the force transducer by a suture (iWorx/CB Science, Inc., Dover, NH, USA). The distal portion of the sciatic nerve was exposed, connected to bipolar electrodes and connected to an electrostimulator (Grass S88; Grass Instruments, Quince, MA, USA). Muscle function was evaluated by measuring the response of isometric contractions, with adjustment for the tension at rest, to obtain the maximum muscle strength (tetanus), using the peak voltage curve caused by the electrostimulator. Muscle strength was recorded and analyzed by Powerlab® 8/30 (ADInstruments Pty Ltd, Colorado Springs, CO, USA).

### Histological analysis

The animals were euthanized and perfused with an intravascular injection of PBS. The muscles were then removed and washed with PBS to wash out the blood. The quadriceps and gastrocnemius muscles were weighed on an analytical balance.

The tissues were fixed in 4% paraformaldehyde for 48 hours, dehydrated and embedded in paraffin. Sections of 4 μm were obtained and used for staining with either H & E to determine the degree of muscle regeneration and the prevalence of adipocytes and infiltrated cells or Picrosirius Red to determine the degree of fibrosis. Other sections were collected on glass slides coated with poly-l-lysine and subjected to immunohistochemistry using anti-alpha-actin (1:50) (clone 1A4; Dako A/S, Glostrup, Denmark) to mark smooth muscle cells and subjected to staining with biotinylated lectin *Griffonia* (bandeiraea) simplicifolia I (Vector Laboratories, Peterborough, UK) to mark the endothelium and activated macrophages, followed by incubation with streptavidin peroxidase (1:100) (Sigma-Aldrich) and detection with chromogen diaminobenzidine.

The images obtained using an optical microscope (Olympus BX60, Shinjuku, Japan) were analyzed digitally. Morphometric analyses of skeletal muscle tissue were performed on each slide, and at least 10 fields of lesions were captured to evaluate necrosis, apoptosis, muscle regeneration, fibrosis and angiogenesis using Image Pro Plus (Media Cybernetics, Rockville, USA).

### Statistical analysis

GraphPad Prism software (Version 5.01, La Jolla, USA) was used for all analyses. The VEGF gene expression was analyzed using the Student *t* test and other expression by one-analysis of variance ANOVA followed by *post-hoc* Bonferroni test. *P* <0.05 was considered statistically significant.

## Results and discussion

The different rate of ischemic recovery between C57/BL6 mice and BALB/c mice is a well-known phenomenon [33-35]. Most animal models of peripheral arterial disease utilize the BALB/c strain because the C57/BL6 strain has a rapid recovery after ischemic injury. This rapid recovery is caused by a genetic difference in the expression of angiogenic factors on chromosome 7 [34]. The genetic difference leads to the increased expression of VEGF (both basal and after ischemia) and TNFα, greater vessel collateralization and a higher rate of angiogenesis in C57/BL6 mice compared with BALB/c mice.

MSCs can secrete significant amounts of cytokines and growth factors [26,40], which promote new vessel formation and remodeling of injured tissues. These observations led to the use of these cells for preclinical studies and clinical trials to treat ischemic limbs and hearts [29,41-43]. MSCs are present in almost every organ and tissue of the body [37], but the MSCs from the bone marrow and adipose tissue are most commonly used for cell therapy because of their abundance and easy large-scale production. However, different results were obtained when MSCs from fat and bone marrow were utilized to treat ischemic tissues [44], showing that these cells may act differently depending on their origin and the microenvironment where they were injected [31,45]. Understanding the quality of MSCs therefore becomes an essential step prior to cell therapy. Because of the genetics of C57/BL6 mice favors the promotion of strong angiogenesis, the MSCs from this strain would be expected to provide greater benefit for the treatment of limb ischemia than MSCs from BALB/c mice.

**Figure 1:**
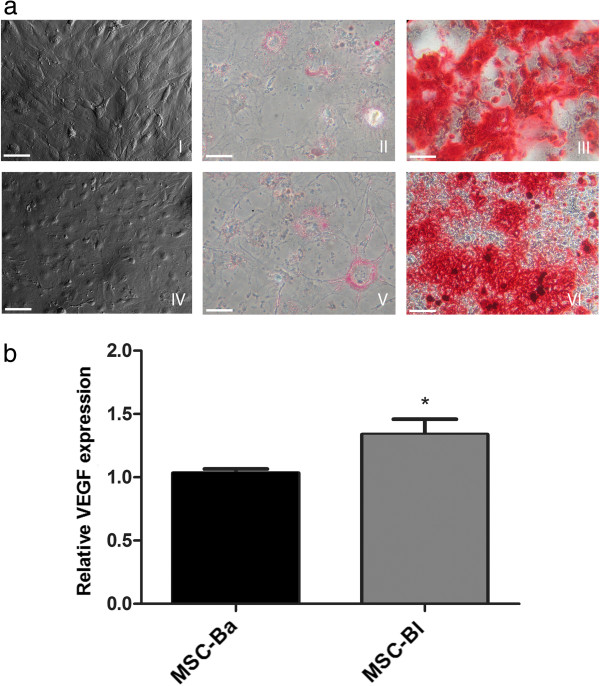
**Characterization of bone marrow mesenchymal stem cells. (a)** Differentiation assay for bone marrow mesenchymal stem cells (MSCs): ischemic animals treated with MSCs obtained from C57/BL6 mice (MSC-Bl; I) and ischemic animals treated with MSCs obtained from BALB/c mice (MSC-Ba; IV). Adipogenic differentiation was evaluated by the formation of lipid vacuoles stained with Oil Red (II, V). The formation of mineralized matrix that stains with Alizarin Red provided evidence of osteogenic differentiation (III, VI). **(b)** Vascular endothelial growth factor (VEGF) gene expression analysis. Comparison of expression between MSC-Bl and MSC-Ba was performed by quantitative real-time PCR. *P <0.05. Bar: 50 μm.

To test this hypothesis, mesenchymal cells were initially extracted from the bone marrow of the femur and tibia of BALB/c and C57/BL6 mice and characterized by differentiation into adipocytic and osteogenic cells (Figure 1a). Differences were noted between MSCs from these two strains during culture maintenance: the growth rate of MSCs derived from C57/BL6 mice (0.8 duplications/day) was higher than that of MSCs derived from BALB/c mice (0.4 duplications/day). As the growth of these cells depends on the presence of various growth factors, this difference may indicate the higher production of cell growth factors by C57/BL6, which also suggests that C57/BL6 MSCs may bring a better therapeutic effect for treating ischemia. The different characteristic of MSCs from both mice is expected because several studies have shown differences in the characteristics of the MSCs according to the tissue and species from which they were extracted [37,46,47].

As commented above, one very important property of MSCs is their capacity for promoting angiogenesis, and VEGF is the main growth factor for this process. Comparing VEGF expression by MSCs of both mice by real-time RT-PCR clearly showed higher VEGF expression by MSCs from C57/BL6 mice than from BALB/c mice, and these data support the hypothesis that the use of MSCs from C57/BL6 mice may yield a better outcome for the treatment of ischemia than MSCs from BALB/c (Figure 1b).

To test our hypothesis, we used a model of limb ischemia that was induced by the complete removal of the femoral artery and the closing of its branches (deep femoral, epigastric and popliteal arteries). According to Goto and colleagues, this model is the best representative model for PAD because it causes severe, stable and uniform ischemia, which is essential to evaluate the effect of the therapy [48]. The mouse strain BALB/c was chosen for the ischemic model because it has a worse response to ischemic induction, with a higher rate of necrosis in the limbs, as demonstrated by Masaki and colleagues [49], and represents a better model for limb ischemia. However, very important to note is that this is not a chronic ischemia model, and is not yet available in the mouse. Because of the different physiology and size of diverse animals, it is very difficult to establish a chronic ischemia model in small rodents.

To treat the ischemic limb of the BALB/c mice, MSCs were injected 5 days after the induction of ischemia. This time was chosen because injecting the MSCs in the first days after injury led to worse results; the rate of necrosis worsened, and many mice suffered amputation (data not shown). The surgical procedures probably caused an acute inflammatory reaction, which attracted neutrophils and macrophages and, consequently, growth factors and proteases were produced locally. Even though MSCs have immunosuppressant properties, these cells also produce growth factors and vessel dilators such as VEGF and nitric oxide, respectively [11,50], to promote angiogenesis and tissue repairing. Excess VEGF and nitric oxide in a tissue is known to be capable of forming unstable and malfunctioning vessels, which can lead to vessel disruption and edema [51,52]. The choice of an adequate moment for cell injection into ischemic tissue for therapy is therefore one of the important points to be considered in cell therapy.

Regarding the injection of MSCs from C57/BL6 mice into the BALB/c limb, there should be no immune response because of the immunosuppressive properties of these cells [53]. This fact is already well established, and MSCs have been used for immunosuppression in several clinical studies [54-56].

**Figure 2:**
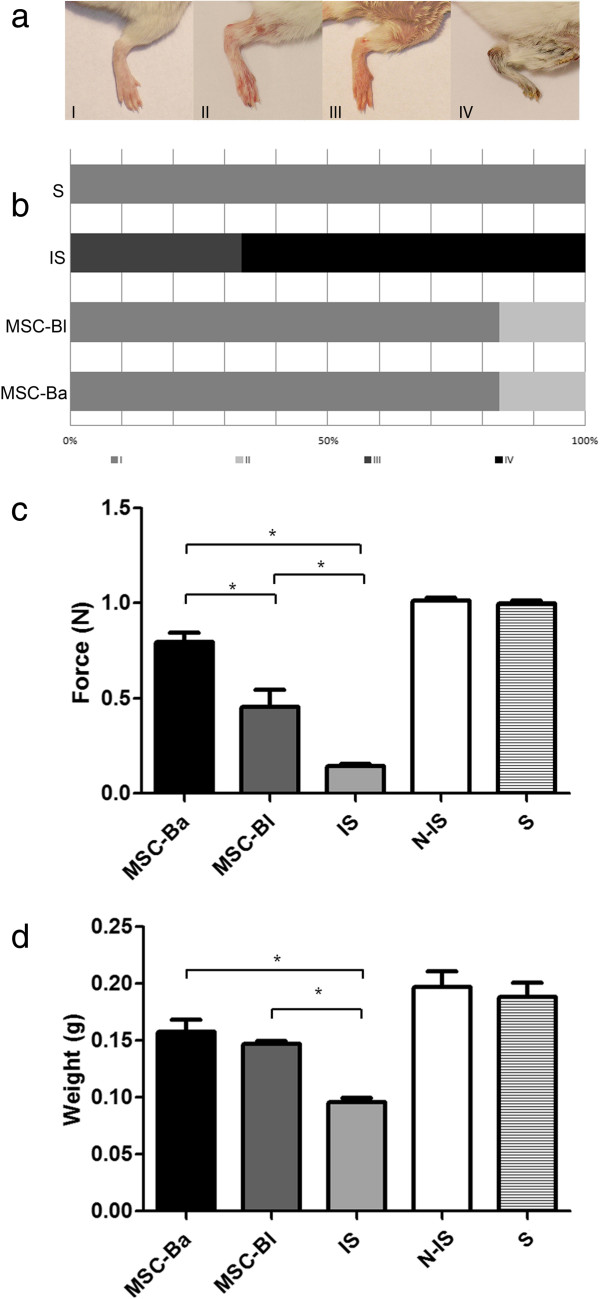
**Therapeutic assessments of the ischemic limbs. (a), (b)** Limb quality was evaluated visually according to the degree of necrosis. Gastrocnemius muscle force **(c)** and mass **(d)** were determined after 30 days of cell therapy. Is, ischemic group; MSC-Ba, ischemic animals treated with mesenchymal stem cells (MSCs) obtained from BALB/c mice; MSC-Bl, ischemic animals treated with MSCs obtained from C57/BL6 mice; N-IS, nonischemic group; S, sham-operated group. *P <0.05, *n* = 6 per group.

The effect of acute ischemia was evaluated visually, which is an easy and non-invasive method. The visual observation was performed for 30 days, which is a sufficient period for the formation of mature vessels and tissue recovery [32,38,39,49]. During this time, the ischemic and untreated mice developed more advanced necrosis; approximately 70% of them reached grade IV necrosis, and the remaining mice developed grade III necrosis (Figure 2a,b). However, the majority of animals treated with MSC-Bl or MSC-Ba showed no advanced necrosis; only 16.7% of the animals developed grade II necrosis (Figure 2a,b). These data indicate that MSCs are highly beneficial for ischemia regardless of their origin.

**Figure 3:**
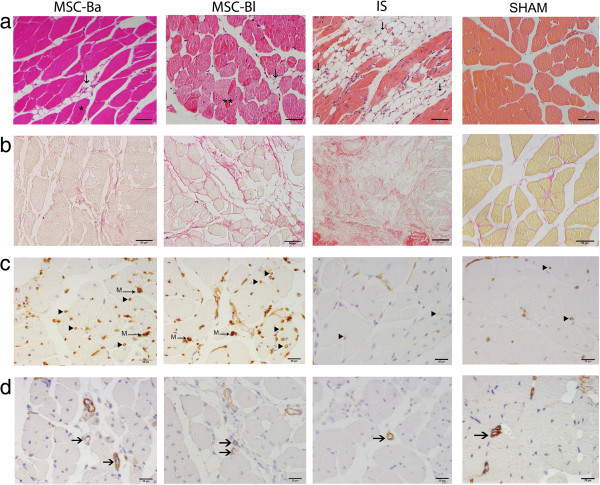
**Histological analysis of limb muscles.** Gastrocnemius muscles were collected after 4 weeks of cell therapy. Tissue samples were stained with **(a)** H & E to assess regenerative (*), necrotic (**) and normal areas and adipocyte infiltration (↓), **(b)** Picrosirius red to fibrotic area (stained in red), **(c)** lectin Griffonia and **(d)** alpha-actin antibody to capillary (arrowheads) density and mature vessels (arrow), respectively. High concentration of leucocytes can be found in necrotic area (**). Macrophages are marked ‘M’ and vessels are marked brown. IS, ischemic group. Bar = 50 μm **(a, b)** and 20 μm **(c, d)**. *n* = 10 fields of lesions area per animal. Is, ischemic group; MSC-Ba, ischemic animals treated with MSCs obtained from BALB/c mice; MSC-Bl, ischemic animals treated with mesenchymal stem cells (MSCs) obtained from C57/BL6 mice; S, sham-operated group.

**Figure 4:**
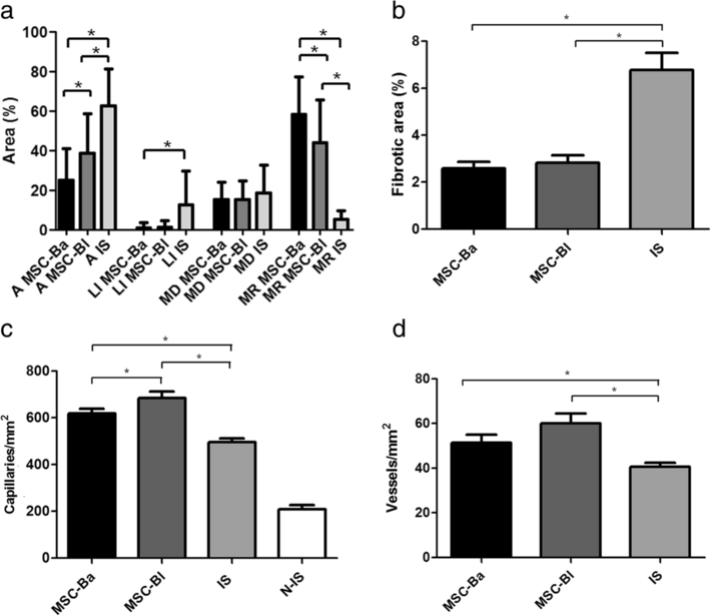
**Morphometric analyses of limb muscles.** From Figure 3, **(a)** necrotic, regenerative and normal areas, **(b)** fibrotic area, **(c)** capillary density and **(d)** matured vessels were determined. More than 50 fields of lesions were counted for each group. A, adipocyte; Is, ischemic group; LI, Infiltrated leukocyte; MD, muscle degeneration; MR, muscle regeneration; MSC-Ba, ischemic animals treated with MSCs obtained from BALB/c mice; MSC-Bl, ischemic animals treated with mesenchymal stem cells (MSCs) obtained from C57/BL6 mice. *P <0.05.

To assess muscle function, which is a very important parameter to evaluate the angiogenic and myogenic effects of cell therapy, muscle strength testing was performed in the gastrocnemius muscle 30 days after treatment. The ischemic animals showed a dramatic reduction in force, which fell from 0.99 N in sham-operated mice to 0.14 N in ischemic mice, while mice treated with MSC-Bl reached 0.45 N, and the animals treated with MSC-Ba experienced the largest recovery, reaching 0.79 N (Figure 2c). However, no significant difference of muscle mass was found between these two treated groups: the treated groups recovered 79% (MSC-Ba) and 74% (MSC-Bl) of their muscle mass compared with the nonischemic mice (Figure 2d). The stronger muscle force observed in mice treated with MSC-Ba must therefore be related to the less adipocytes and greater regeneration of muscle fibers in comparison with the MSC-Bl group (Figures 3 and 4a).

To better understand the effect of MSCs in ischemic muscles at the cellular level, the gastrocnemius muscle was stained with H & E for a histological evaluation of the ischemic tissues in terms of the presence of adipocyte, leukocyte infiltration, cellular degeneration and regeneration. The infiltration of leukocytes was significantly reduced in treated animals compared with ischemic animals, varying from 12.8% to 1.4% and 1.1% in the groups treated with MSC-Bl and MSC-Ba, respectively. The amount of adipocytes was also reduced from 62.8% in the ischemic group to 38% and 26% for groups treated with MSC-Bl and MSC-Ba, respectively. Muscle regeneration had a large increase in the treated groups compared with ischemic group, from 5% in the ischemic group up to 57% in the group treated with MSC-Ba and 44% in the group treated with MSC-Bl. Conversely, cellular degeneration remained similar between the ischemic group (18.9%) and the groups treated with MSC-Bl (15.5%) and MSC-Ba (15.2%) (Figures 3 and 4a). These results indicate that treating ischemia with MSCs promoted a remarkable regeneration of muscle tissue regardless of their origin, which confirms the visual assessment results but contradicts our initial hypothesis because there was no significant difference between the treated groups.

To assess whether the treatment promoted neovascularization, the sectioned slides were stained with Griffonia lectin, which marks the endothelium of blood vessels (and activated macrophages, which can be visually differentiated), and anti-alpha-actin, which marks alpha-actin of vessel smooth muscle cells. Staining with Griffonia showed a significantly increased number of capillaries in both treated groups (684 and 619 vessels/mm^2^ in the MSC-Bl and MSC-Ba groups, respectively) compared with the ischemic group (497 vessels/mm^2^) (Figures 3 and 4b), but there was no significant difference between the treated groups. Immunohistochemistry for alpha-actin also showed a similar pattern; the ischemic group had 40.7 vessels/mm^2^, and the treated group had higher densities (57.8 and 51.8 vessels/mm^2^ in the MSC-Bl and MSC-Ba groups, respectively) (Figures 3 and 4c). These results show increased vessel densities after treatment with MSCs, but no difference was observed between the MSC-Bl and MSC-Ba groups.

Finally, a histological evaluation of fibrosis was performed with Picrosirius red dye, which stains collagen. In all treated groups, a remarkable reduction in the fibrotic areas was observed, decreasing from 6.8% in the ischemic group to 2.8% and 2.6% in groups treated with MSC-Bl and MSC-Ba, respectively (Figures 3 and 4d). This reduction is most probably due to mesenchymal cells, which can prevent collagen deposition and the formation of fibrosis [57] by secreting hepatocyte growth factor [58]. There was no difference between the two treated groups, indicating that the genetic difference between the two strains of mice also did not influence this parameter.

## Conclusion

Our results collectively demonstrate the important role of MSCs in the recovery of ischemic tissues, regardless of the genetic background. These cells were able to prevent necrosis, decrease inflammatory and adipocytes, increase muscle strength and mass, decrease fibrotic area and promote neovascularization by increasing the number of capillaries and larger vessels compared with the untreated group. However, contrary to our initial hypothesis, the treatment with MSCs from C57/BL6 mice did not produce better angiogenic results than the treatment with MSCs from BALB/c mice. In terms of myogenesis, the MSCs from BALB/c mice promoted significantly better outcome.

These observations indicate that the amount of growth factors initially produced by the MSCs is most probably not the main factor that promotes more angiogenesis and brings better long-term tissue regeneration. As previously suggested, perhaps the main role of MSCs in the injured tissue is to recognize the needs of the microenvironment and to reprogram local cells to replace their own damaged tissues, releasing cytokines that modulate inflammation and immune system [59]. The similar results obtained with MSCs from BALB/c mice and C57/Bl6 mice, which have a clear genetic difference in term of angiogenesis, when treating ischemic tissue corroborates strongly this idea.

## Abbreviations

DMEM: Dulbecco’s modified Eagle’s medium; DMEMc medium: DMEM supplemented with 2 mM glutamine, 200 U/ml penicillin, 200 μg/ml streptomycin and 10% fetal bovine serum; H & E: hematoxylin and eosin; IL: interleukin; MSC: mesenchymal stem cells; PAD: peripheral arterial disease; PBS: phosphate-buffered saline; PCR: polymerase chain reaction; RE: reverse transcription; TNF: tumor necrosis factor; VEGF: vascular endothelial growth factor

## Competing interests

The authors declare that they have no competing interests.

## Authors’ contributions

FFdC participated in all experiments and helped to draft the manuscript. LM carried out ischemic induction and force tests. PKMM participated in MSC culture and analysis. RSS carried out real-time PCR and its analysis. SWH conceived of the study, and participated in its design and coordination and helped to draft the manuscript. All authors read and approved the final manuscript.

## Authors’ information

FFdC graduated in pharmacy and biochemistry and has an MSc in molecular biology. LM graduated in biomedicine and is currently an MSc student. PKMM and RSS graduated in biomedicine and are currently PhD students in molecular biology. SWH has a PhD in biochemistry and is a professor of biophysics.
